# See, Feel, Taste: The Influence of Receptacle Colour and Weight on the Evaluation of Flavoured Carbonated Beverages

**DOI:** 10.3390/foods7080119

**Published:** 2018-07-26

**Authors:** Line Ahm Mielby, Qian Janice Wang, Sidsel Jensen, Anne Sjoerup Bertelsen, Ulla Kidmose, Charles Spence, Derek Victor Byrne

**Affiliations:** 1 Department of Food Science, Faculty of Science and Technology, Aarhus University, Kirstinebjergvej 10, DK-5792 Aarslev, Denmark; lineh.mielby@food.au.dk (L.A.M.); annesbertelsen@food.au.dk (A.S.B.); ulla.kidmose@food.au.dk (U.K.); derekv.byrne@food.au.dk (D.V.B.); 2 Crossmodal Research Laboratory, Department of Experimental Psychology, Oxford University, New Radcliffe House, Oxford OX2 6BW, UK; charles.spence@psy.ox.ac.uk; 3 Carlsberg Breweries A/S, J. C. Jacobsens Gade 4, DK-1799 Copenhagen, Denmark; sidsel.jensen@carlsberg.com

**Keywords:** crossmodal correspondences, weight, colour, sweetness, carbonation, mediation, product design

## Abstract

A study was designed to assess whether the individual and combined effects of product-intrinsic and product-extrinsic factors influence the perception of, and liking for, carbonated beverages. Four hundred and one participants tasted samples of one of three flavours (grapefruit, lemon, or raspberry) of carbonated aromatised non-alcoholic beer. The beverages were served in receptacles that differed in terms of their colour (red or black) and weight (lighter—no added weight, or heavier—20 g weight added). Each participant received the same beverage in each of the four different receptacles, and rated how much they liked the drink. They also evaluated the intensity of each beverage’s sweetness, bitterness, sourness, and carbonation. The results revealed a significant influence of the colour of the receptacle on perceived carbonation, with the beverages tasted from the red receptacles being rated as tasting more carbonated than when served in black receptacles. In terms of flavour, the participants liked the raspberry beverage significantly more than the others, while also rating it as tasting sweeter and less bitter than either of the other flavours. Furthermore, there was a more complex interaction effect involving the weight of the receptacle: Specifically, the perceived bitterness of the beverage moderated the relationship between the receptacle weight and the perceived carbonation. At high levels of bitterness, the drinks were perceived to be more carbonated when served from the heavier receptacle as compared to the lighter one. These findings highlight the complex interplay of product extrinsic and intrinsic factors on the flavour/mouthfeel perception and preference for beverages, and stress the importance of taking both internal product development and external packaging into account in the design of health-oriented beverages.

## 1. Introduction

Human perception and preference for food and beverage products are undoubtedly major determinants of their success in the marketplace (e.g., [1,2]). The multisensory experience of a food product, and thus product choice, is a multifactorial and dynamic phenomenon. A vast body of research now supports the view that both food product-intrinsic and food product-extrinsic factors (such as environmental or packaging cues) play an important role in the perception and acceptance of what we choose to eat and drink. However, it is still unknown as to how these ever-present intrinsic and extrinsic factors interact. Here, we focus on how the interaction between the flavour of carbonated beverages on the one hand, and the colour and weight of the serving receptacle on the other, can influence product preference, and the perception of basic tastes and carbonation.

### 1.1. Crossmodal Influence of Aroma on Basic Taste Perception

In terms of product-intrinsic factors, the aroma (perceived both ortho- and retronasally), colour, and oral-somatosensory texture of food and beverage items have, among others, been found to affect sweetness perception (for reviews, see [3,4,5,6,7]). 

Aromas can be used to modify taste perception (see [3] for a review), Interestingly, “sweet” is one of the most frequently used descriptors for odours, even though sweetness itself is, by definition, not an odour, but a basic taste [8]. This is a learned behaviour, given that the majority of the aromas that are typically associated with sweetness are those related to previous instances of co-exposure in food products with a dominant sweet taste [3]. For example, aromas such as caramel and strawberry have both been shown to increase the perception of sweetness in Western participants [4,9,10]. In a study of Frank and Byram [9], four sub-studies were conducted looking at the perception of sweetness and saltiness in different food matrices: sucrose-sweetened whipped cream with strawberry aroma, sucrose-sweetened whipped cream with peanut butter aroma, salted whipped cream (with sodium chloride) with strawberry aroma, and finally sucrose-sweetened whipped cream with strawberry aroma, evaluated with the participant’s nose pinched shut. These researchers found that strawberry aroma enhanced the perception of sweetness; that an aroma’s ability to enhance sweetness is aroma-dependent; and that an aroma’s ability to enhance taste is taste-dependent. Finally, the authors concluded that the influence of the strawberry aroma on sweetness perception was caused by the perception of the aroma orthonasally through the nose, rather than retronasally via the mouth. However, other researchers have subsequently demonstrated that both orthonasal and retronasal enhancements effect certain aromas for tastes such as sweetness [11,12] (see [5] for a review).

### 1.2. Influence of Container Colour and Weight on the Perception of Basic Tastes and Flavour

A growing body of scientific research shows that people systematically associate different colours of foods and beverages (regardless of whether they are found in the food itself or in the food presentation/packaging), with specific basic tastes (see [13,14] for reviews). In one early study, O’Mahony [15] reported that U.S. participants consistently matched the colour red to sweet tastes, yellow to sour tastes, and white to salty tastes. The impact of particular colours on the perception of specific tastes has been repeatedly demonstrated over the years. Specifically, in terms of sweetness, red-coloured drinks have been found to enhance the detection of sweetness [16], expectations of sweetness [17], and perceived sweetness intensity [18,19,20,21]. However, in terms of the sensitivity to sweet taste, Maga [22] did not observe any effect from the colour red on taste detection thresholds. Rather, the colour red decreased people’s sensitivity to bitter tastes. Going beyond the colour of the drink itself, pink receptacles are more closely associated with sweetness than are transparent receptacles [23], and popcorn tasted from red bowls is reported to be approximately 15% sweeter as compared to popcorn from a white bowl [24]. Additionally, Woods and colleagues [25] found that pale pink alone or as part of a colour pair communicated the sweet taste more effectively than did any other colour. That said, it is worth noting that in all of the early studies between colour and taste, participants were restricted to the set of four or five basic tastes—sweet, salty, sour, bitter, and possibly also umami. That these studies focused on a basic taste framework may be seen as somewhat limited in perspective, given that red colour is, in addition to being matched to sweetness, also associated with other sensory attributes of food such as spicy [26,27], and even carbonation [28,29]. Context presumably plays an important role here, such that a red-coloured salsa might be seen as being more spicy, whereas a red-coloured beverage would likely be rated as being sweeter.

Compared to colour, the influence of container weight on food perception has not been studied extensively. Piqueras-Fiszman and colleagues [30] tested consumers tasting identical yogurts from bowls that only differed in terms of their weight. The yogurt samples from the heaviest bowls were rated as being more dense, more highly preferred, and the participants expected them to be more expensive than those from lighter bowls; however, there was no significant difference in terms of the perceived flavour intensity. In a follow-up study, the weight of the bowl was also found to influence the expected satiety, with food served in the relatively heavier of two containers expected to be more satiating [31]. The influence of the weight of packaging on increasing the desire for consumption and willingness to pay was also documented by [32], using boxes of chocolates as well as cans of soft drinks, some with added weights. The authors proposed a model whereby the weight of the packaging influenced consumer purchase intentions via the mediating effect of raising the perceived flavour intensity (see [33], for evidence that perceived fragrance intensity of bottles of liquid soap is also influenced by their weight). 

Finally, there is some evidence that extrinsic factors can influence the perception of carbonation. Carbonation is a type of oral-somatosensory texture, which, from a physiological point of view, is typically perceived as having an acidic taste, presumably because carbon dioxide is detected by the sour-sensing cells on the tongue [34]. In addition, dissolved carbon dioxide in water forms a small amount of carbonic acid in equilibrium [35], which can taste mildly sour. In a study on the relationship between the level of carbonation and container weight, Maggioni and her colleagues [36] found that sparkling mineral water sampled from heavier receptacles was perceived as less pleasant and more carbonated than the same samples from lighter receptacles. Furthermore, Risso and her colleagues [29] went on to demonstrate that mineral water at various carbonation levels tasted more carbonated when tasted from red or blue receptacles, as compared to when tasted from a white receptacle.

### 1.3. The Food Matrix as a Moderating Factor

In terms of interactions between food-intrinsic factors, it has been demonstrated that taste-aroma interactions are moderated by the nature of the food matrix in question. Labbe and his colleagues [37] tested the taste enhancement effects of cocoa and vanilla flavouring in cocoa and caffeinated milk. They found that, in the cocoa beverage, cocoa flavouring led to an enhancement of bitterness, and vanilla flavouring enhanced sweetness. However, when it came to the relatively less familiar caffeinated milk product, the addition of vanilla flavouring unexpectedly enhanced bitterness instead of sweetness. Elsewhere, Alcaire et al. [38] reported that while an increase in vanilla flavour in a dairy dessert product had a minor effect on sweetness enhancement, the combination of increased vanilla concentration, together with higher starch concentration, led to an increase in vanilla flavour intensity, as well as an increase in perceived sweetness. This was presumably due to the thickened viscosity of the dessert product from the addition of starch. Hewson et al. [39] investigated the effect of varying types and levels of sugars (glucose and fructose) and acids (citric and lactic acid) on flavour and taste perception in a model citrus-flavoured beverage. Despite there being no instrumentally measured effect on aroma release and viscosity, they found that flavour perception increased upon the addition of tastants, but that glucose- and fructose-containing beverages showed different profiles even though the levels of glucose and fructose used were not perceptibly different in terms of sweetness.

There is also evidence that the flavour of the foodstuff itself can moderate the influence of extrinsic factors such as colour, shape of receptacle, or background sound [26,40,41]. For instance, adding red colouring to tomato salsa samples has been shown to enhance their perceived spiciness, but only when the samples were somewhat spicy to begin with [26]. Similarly, Wang and colleagues [41] recently demonstrated that a custom-composed spicy soundtrack had similar spiciness enhancement properties; but once again, the auditory enhancement effect was only present for samples of spicy, but not mild, salsa.

As seen above, both product-intrinsic and product-extrinsic factors affect how we perceive and affectively respond to foods and beverages. Due to the dynamic correlation between all factors, they should ideally all be taken into consideration in consumer studies on food and beverages. However, researchers most often tend to focus on either intrinsic or extrinsic factors. In fact, this trend is also somewhat reflected in the organisational structure of many large food companies where Research & Development (R&D), which is in charge of food-intrinsic properties, typically sits far away from, and actually has little interaction with, the marketing department, who may be responsible for product packaging.

### 1.4. Aims and Hypothesis

The current study was designed to assess whether and how the individual and combined effects of intrinsic (flavour) and extrinsic (colour and weight of serving receptacle) factors influenced consumer perception and preference for three differently flavoured carbonated beverages (raspberry, lemon, and grapefruit). First, the effects of intrinsic and extrinsic factors were evaluated across all flavoured beverages. Next, potential interactions between intrinsic and extrinsic factors were identified and further analysed using moderation analysis. The underlying hypothesis was that all factors studied—both intrinsic and extrinsic—could influence how consumers perceive and affectively respond to the carbonated beverages. More specifically, given the evidence reviewed above, we would expect perceived sweetness to be influenced by the product flavour (with the berry flavour being perceived as sweeter than the two citrus flavours) and receptacle colour (with red being associated with greater sweetness). Additionally, we would also expect perceived carbonation to be influenced by both receptacle colour and weight (with red colour and heavier weight both being associated with increased carbonation). Furthermore, it is possible that the effect of extrinsic factors could be moderated by the intrinsic properties of the beverages. The study was performed using commercially available products rather than model systems, in order to increase the ecological validity of the study.

## 2. Materials and Methods

### 2.1. Experimental Overview

**Figure 1:**
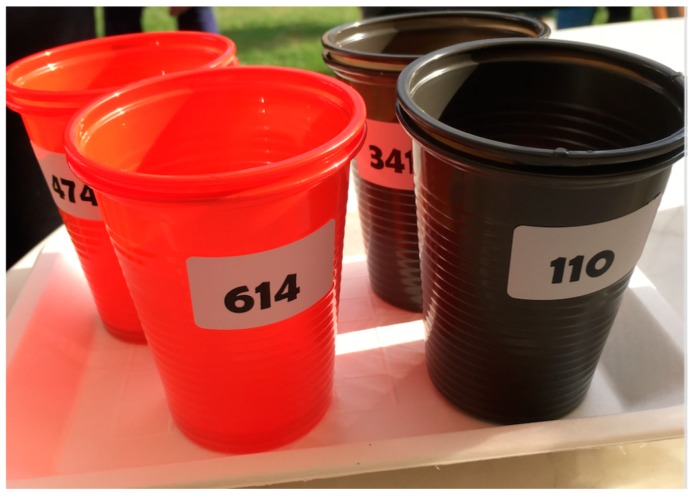
Experimental setup. The particular set of 3-digit numbers shown in the picture corresponds to the group of participants who received the grapefruit-flavoured beverages.

The effect of receptacle colour (red, black) and weight (light, heavy) on consumers’ perception and beverage preferences was tested for three differently-flavoured carbonated beverages in a mixed-model design (Figure 1). Flavour was a between-participants factor, whereas receptacle colour and weight were within-participant factors. More specifically, one participant group tested the effect of different receptacles on grapefruit-flavoured beverages, a second group tested the effect on lemon-flavoured beverages, and a third tested the effect on raspberry-flavoured beverages.

### 2.2. Participants

A total of 401 adult participants (125 males, total mean age of 32.2 ± 14.3 years) were recruited at Aarhus University’s stand at a large food festival over a two-day period in Aarhus, Denmark. The participants were randomly divided into three groups, where each group tasted one specific flavour variant of the test beverage. The three groups were not significantly different in terms of their gender distribution and age (see Table 1, for gender, χ^²^(2) = 3.74, *p* = 0.15; for age, *F*(2,398) = 1.16, *p* = 0.31). All of the participants were recruited at the actual test site. The participants were informed that their participation was completely voluntarily and that they could choose to withdraw at any time. Additionally, they were informed that all data would be treated anonymously. After giving their written consent, the participants took a seat at a table within the stand. Since all data were collected anonymously and no potentially harmful procedures were used, ethical approval was not sought for the execution of this study. 

### 2.3. Stimuli

#### 2.3.1. Beverages

Three flavour variants (grapefruit, lemon, and raspberry) of Tourtel^®^ Twist, (Copenhagen, Denmark), a non-alcoholic beer which is available on the French market but unfamiliar to Danish consumers, were used in the experiment. As with a regular lager beer, Tourtel^®^ Twist, when poured, has a pale head and is carbonated. The product comes in different flavours as a result of added fruit concentrates and natural aromas, and is described by the distributor to be “without any noticeable bitterness”. The colours of the beverages were all pale and cloudy (due to the presence of yeast particles), ranging from light yellow (Tourtel^®^ Twist Lemon), to light orange (Tourtel^®^ Twist Grapefruit) and finally dusty pink (Tourtel^®^ Twist Raspberry). There were minor differences in the total carbohydrate, sugar, protein, and fat content between the three flavour variants (Table 2). However, the difference in sugar level is less than the just-noticeable difference (JND), at 5.1 g/100 mL [42]. The beverages were served at room temperature in 30-mL portions into different receptacles (Section 2.3.2), and were poured immediately before tasting to avoid differences in the level of carbonation. All receptacles carried three digit codes. 

#### 2.3.2. Beverage Receptacles

For all three sub-studies, four different types of receptacles were used with two levels of colour (red or black) and two levels of weight (no weight added or extra weight added). Regular 200 mL red and black plastic cups (ILIP, Miro series, Bazzano, Italy) were used. The colour red was chosen for its positive association with sweetness [24]. While pink is also associated with sweetness [23,25,43], red cups were chosen because they masked the colour of the actual beverage better than pink. Since we were interested in masking the colour of the actual beverage, black cups were chosen as a “control” rather than white (Note here that white and black are also both associated with basic tastes, white with saltiness [15], and black with bitterness [44]). In order to mask the addition of the weight, two cups were stacked, one on top of another, for all four receptacle types. In the weight-added versions, a 20 g metal disc (the same weight added as in [36]) with dimensions 13 × 37 × 3 mm (tj-bolte.dk, Jerslev, Denmark) was placed in between each pair of stacked cups.

### 2.4. Procedure

Each participant was randomly assigned to one of the three beverage flavours. The experimenter provided the participants with four samples of the specific flavoured beverage served in four different types of receptacles (see Figure 1), water, a paper questionnaire, and a pen. The participants were first given a short verbal introduction to the test. Participants were asked to match the 3-digit identifier given in each trial with the 3-digit identifier labeled on each sample (see Figure 1). For each sample, they were asked to first taste the beverage (they were not required to finish each 30 mL sample), and then rate their overall liking for the beverage, as well as perceived intensities of sourness, sweetness, bitterness, and level of carbonation. All ratings were made on nine-point scales with endpoint categories anchored by “not at all” on the left (corresponding to a value of 1) and “extremely” on the right (corresponding to a value of 9). The liking scale also included a midpoint (corresponding to a value of 5), which was labeled “neither like nor dislike”. To control for presentation bias, four different versions of the questionnaires with differing sample presentation orders were used according to a Latin Square table. All of the participants were offered sweet treats and apples after taking part in the study. The test procedures were pilot-tested amongst the staff at the Aarhus University Department of Food Science before the actual consumer study.

### 2.5. Data Analysis

To provide an overview of the dataset as a whole, Pearson’s correlation coefficients for all flavours in combination with all response variables were calculated (SPSS, version 23.0, IBM Corp., Armonk, NY, USA). To assess the overall effect of receptacle colour and weight on consumer perception and liking for carbonated flavoured beverages, repeated measures of analyses of variance (rm-ANOVAs) were conducted with flavour as a between-participants factor; receptacle colour and weight as the within-participants factors; and rated liking, sweetness, sourness, bitterness, and carbonation as measures. Effect sizes were examined using partial eta squared values. All post-hoc pairwise comparisons were Bonferroni-corrected.

To further understand the nuances of interaction effects, we explored the perceived food tastes as possible moderating factors on the influence of receptacle colour and weight using the PROCESS macro in SPSS [45].

## 3. Results

### 3.1. Product and Ratings Overview

To gain an overview on how the response variables were interrelated, Pearson’s correlation coefficients were generated for all response variables for all flavour variants combined. It showed, as expected, that preference was positively correlated with perceived sweetness, and it was negatively correlated with perceived bitterness and sourness (see Table 3). Furthermore, carbonation was positively correlated with perceived sourness and bitterness, which was in line with previous research that suggested that people perceive carbonation as acidic [34].

### 3.2. Effect of Colour and Weight of Receptacle

**Figure 2:**
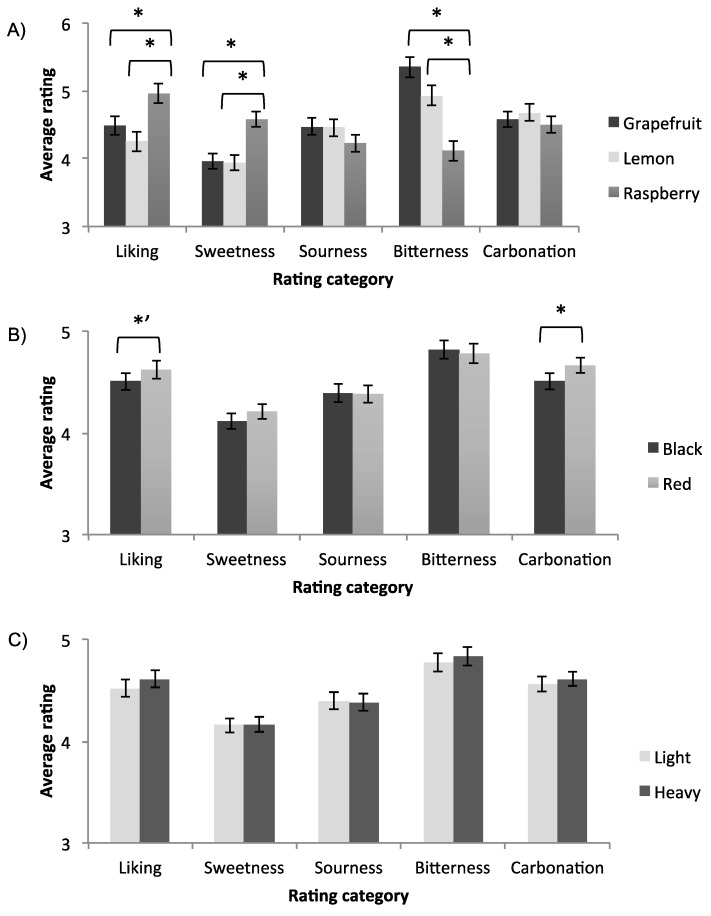
Participants’ average rating scores for each rating category (liking, sweetness, sourness, bitterness, carbonation), factored by (**A**) flavour, (**B**) receptacle colour, (**C**) receptacle weight. The present study used a mixed-model design where flavour was a between-participants factor (*N*_grapefruit_ = 134, *N*_raspberry_ = 132, *N*_lemon_ = 135), and receptacle colour and weight were within-participants factors. All rating categories were measured with 9-point scales (1 = not at all; 9 = extremely), and the overall liking scale had an additional anchor at 5 for “neither like nor dislike”. Error bars indicate standard errors. Asterisks indicate statistical significance (* *p* < 0.05, *’ *p* < 0.10).

Rm-ANOVAs were conducted with flavour as the between-participants factor, receptacle colour and weight as the within-participant factors, and rated liking, sweetness, sourness, bitterness, and carbonation as measures (see Table 4). Average values of the dependent measures are shown in Figure 2. The analysis revealed that there was a significant main effect of the receptacle colour on carbonation, where beverages served in the red receptacle were perceived to be more carbonated than those served in the black receptacle (*M_black_* = 4.51, *Standard Error (SE)* = 0.08, *M_red_* = 4.66, *SE* = 0.07, *p* = 0.02). In addition, there was a significant effect of flavour on preference, sweetness, and bitterness; raspberry was more highly preferred, and it was rated as sweeter and less bitter than grapefruit and lemon. Notably, the effect sizes of the product flavour (*η*_p_^2^ = 0.035, 0.051, 0.086), which are medium-sized according to [46], were larger than the effect size of colour (*η*_p_^2^ = 0.014), which is a small effect.

**Figure 3:**
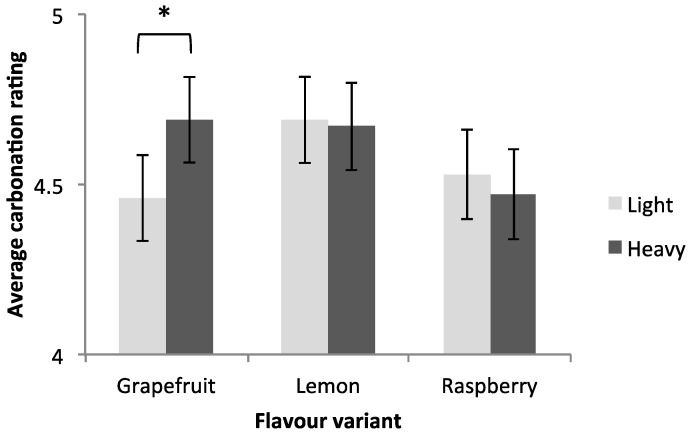
Participants’ average rating scores for carbonation (on a scale of 1−9, with 1 = not at all; 9 = extremely), for each flavour variant, tasted in either light or heavy (with +20 g extra weight) receptacles. Flavour was a between-participants factor (*N*_grapefruit_ = 134, *N*_raspberry_ = 132, *N*_lemon_ = 135), whereas receptacle weight was a within-participants factor. Error bars indicate standard errors. Asterisks indicate statistical significance (* *p* < 0.05).

There was also a trending interaction effect between receptacle weight and product flavour on carbonation (*F*(2,382) = 2.76, *p* = 0.064, *η*_p_^2^ = 0.014). Further univariate ANOVAs revealed that, for the grapefruit-flavoured beverage, receptacle weight had a significant effect on carbonation, where the beverages in the heavier receptacle were perceived as being more greatly carbonated than the beverages served in the lighter receptacle (*M_heavier_(SD)* = 4.69 (0.12), *M_lighter_(SD)* = 4.46 (0.13), *F*(1,31) = 6.05, *p* = 0.015, *η_p_^2^* = 0.044). We did not observe similar influences for weight on carbonation, for the lemon (*p* = 0.79) or raspberry (*p* = 0.53) flavour variants (see Figure 3). Referring to Figure 2A, the grapefruit variant stood out amongst the different flavours as having high levels of bitterness.

**Figure 4:**
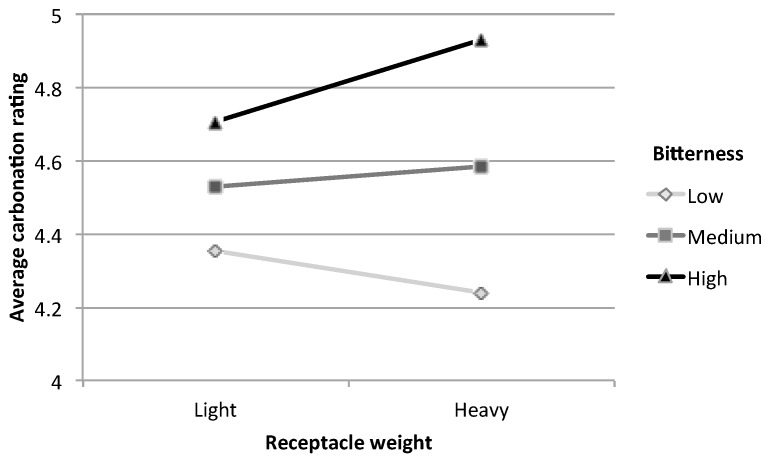
Interaction plot showing participants’ average carbonation ratings for light and heavy beverage receptacles at three levels of perceived bitterness. Low bitterness = 2.61 (one standard deviation below the mean), medium = 4.79 (mean bitterness rating), high = 6.96 (one standard deviation above the mean). Note that at high bitterness levels, beverage tasted from heavier receptacles seem to be more carbonated than from a lighter one.

To test the hypothesis that the perception of carbonation might be a function of both receptacle weight and perceived bitterness, a moderation analysis was conducted. Running the PROCESS macro revealed that the interaction between receptacle weight and perceived bitterness across all three beverages accounted for a small but significant proportion of the variance in terms of perceived carbonation, *R*^2^ = 0.0024, *F*(1,1589) = 3.88, *p* = 0.049; *b* = 0.078, *t*(1589) = 1.97, *p* = 0.049. Furthermore, the interaction plot revealed that for high bitterness levels (defined as one standard deviation above the mean bitterness level), beverages from the heavier receptacles were perceived as being more carbonated than those from light receptacles (see Figure 4).

## 4. Discussion

### 4.1. Effect of Receptacle Colour and Weight on Preference and Perception of Basic Tastes

We first assessed the effect of receptacle colour and weight on beverage preference and perception of basic tastes for all flavour variants in combination. There was no significant influence of receptacle colour or weight on the perception of sweetness, but we did observe a clear influence of these product-extrinsic factors on carbonation. Overall, beverages in red receptacles were perceived as being more carbonated than the same beverages presented in black receptacles. Risso and her colleagues [29] previously obtained similar results with carbonated water, where participants rated mineral water at various carbonation levels as tasting more carbonated when tasted from red or blue receptacles, compared to a white receptacle [28]. The authors proposed a potential explanation for the effect, whereby the receptacle colour might alter the participants’ level of emotional arousal/interest, which, in turn, leads to higher perceived carbonation. This could potentially apply to the present study, since red colours have been shown to increase arousal compared to black (see [47] for a review). The results of the current study extend those of [29], since they demonstrate the effect of the colour of receptacle on the perception of carbonation across carbonated beverages with different flavours. Moreover, the fact that red colour was associated with increased carbonation rather than sweetness might be due to the fact, as mentioned in the introduction, that the influence of colour is context-dependent [26]. Since the beverages were relatively low in sweetness (as observed from the participants’ mean sweetness ratings), red became associated more closely with carbonation than with sweetness. 

According to the literature [24,48], we should have expected to see a significant influence of receptacle colour on sweetness. We did not observe such an effect due to the following potential reasons: (1) any influence of the colour of the receptacle is weaker than the influence of product’s colour itself, (2) the participants recognized the four samples as identical, and (3) the products were not very sweet to begin with (citrus and grapefruit beverages were rated at 4/9 for sweetness, on average). Schifferstein [23] assessed the expected and actual experience of drinking two different liquids, soda or hot tea, in receptacles made from different materials. The participants in Schifferstein’s study associated pink coloured receptacles with higher sweetness ratings than clear glass ones. In our case, it was equally likely that the red receptacles induced expectations of sweetness in the tasters. However, because the beverages were relatively low in sweetness (especially the lemon and grapefruit variants, see Figure 2), it is possible that the tasters experienced a disconfirmation of expectations. In other words, we did not observe any enhanced sweetness, possibly because the beverages were simply not sweet enough to match the expectations set up by red receptacles. 

### 4.2. Effect of Flavour on Liking and Perception, Specifically Sweetness

There was a difference in both beverage preference and taste ratings among the different flavoured carbonated beverages. The results revealed that the product flavours had a significant impact on perceived sweetness, as the raspberry-flavoured drink was perceived as being sweeter than the two citrus-flavoured drinks. Nevertheless, all three flavours contained approximately the same amount of sugar that was not discernably different (ranging from 5.1 to 5.3 g/100 mL, see [42]) and were otherwise comparable in terms of their nutritional content, and did not contain any artificial sweeteners. However, since the drinks were flavoured with fruit concentrates and natural aromas, it is possible that these concentrates contained varying levels of bitter and/or sour compounds, which may have masked the sweetness in the beverage (specifically in the case of the citrus flavours). Bitterness and sourness, after all, have often been found to have a suppressing effect on the perception of sweetness (e.g., [49]). This also supports the fact that the raspberry drink was rated as being less bitter than the two citrus flavours. The raspberry-flavoured beverage was additionally the most preferred variant tested in this study (or rather, at a value of 5/9 it was neither liked nor disliked, whereas the other flavours were somewhat disliked). This result was perhaps to be expected, as humans have an innate and rather persistent preference towards sweet foods [1]. Another contributing factor influencing the perception of sweetness could be the aroma characteristics of the differently flavoured beverages. Certain aromas are consistently reported as “smelling sweet” in the literature, even though sweetness is normally associated with the stimulation of the sense of taste [8]. Additionally, as Stevenson and Boakes [8] have pointed out, the large number of aromas, which are reported to “smell sweet”, are, from a chemical and perceptual point of view, quite different, and thus, this does not explain why they “smell sweet”. Rather, it is more likely that the raspberry-flavoured beverage was perceived to be sweeter because raspberries are naturally associated with sweeter tastes (or less sour and bitter tastes) compared to grapefruit and lemon, which are commonly known for their more bitter and sour sensory characteristics, respectively. In order to elucidate the underlying reason for the higher sweetness ratings for the raspberry-flavoured beverages, we should ideally have made the participants rate the sweetness of the beverages under two different conditions—as an orthonasal sniff-only condition, and as a nose-pinched taste-only condition. Otherwise, a descriptive analysis using a trained sensory panel would have also given us more information on the overlying reasons for these results.

### 4.3. Combined Effects of Intrinsic (Flavour) and Extrinsic Factors (Colour, Weight) on Liking and Perception

Besides a study on different coloured yoghurts sampled with spoons of different colours [50], a study on the effect of receptacle shape on taste perception of two different beverage types [40], and the study on different levels of carbonated water served in different coloured receptacles [29], the present study is one of the first of its kind to focus on the combined effects of intrinsic and extrinsic factors on beverage preference and flavour perception. It was therefore interesting to determine whether there were any significant interactions, particularly between product flavour (intrinsic) and receptacle weight/colour (extrinsic).

The moderating effect of product bitterness on how receptacle weight influences perceived carbonation is a good example of why product-intrinsic factors should be taken into account when designing crossmodal studies (and products). We found that participants who drank from heavier receptacles perceived more carbonation (in agreement with findings from [36]), but only when the beverage was perceived to be quite bitter. One possible explanation for this is that the heavier receptacles enhanced the flavour intensity of the beverages in general [32,33], and greater perceived bitterness was associated with greater carbonation, because there is a strong correlation between bitterness and carbonation (see Table 2). Of course, this leaves us with the question of why we did not see a similar moderating influence of sourness, as sourness is also associated with carbonation [34]. One explanation is that participants may simply have confused sourness with bitterness, as this is a common occurrence amongst the general population [51,52]. Alternatively, however, it is possible that the drinks were simply not sour enough, and that people’s flavour perception—especially for the grapefruit- and lemon-flavoured beverages—was instead dominated by bitterness. It would be intriguing to repeat the study with obviously sour but not bitter beverages, where we should expect to observe a similar effect of receptacle weight on carbonation for the sour drinks. 

Along with calculating the significant differences between the intrinsic (flavour) and extrinsic (colour and weight of receptacle) factors, effect sizes were also calculated. This made it possible to compare which of the factors were most influential in terms of the participants’ ratings of the stimuli. According to the results of the combined flavour model, the effect size of the product flavour was medium, compared to the relatively small effect size of receptacle colour. For this particular study, this means that the intrinsic factor of product flavour was more influential (in terms of explaining a greater amount of variance in participants’ responses) as compared to the extrinsic factor of receptacle colour. It should also be clarified here that the medium effect size of product flavor was found for ratings of overall beverage preference, sweetness, and bitterness, where receptacle colour did not have a significant influence. On the other hand, for the ratings of carbonation intensity, the effect size of the receptacle colour was greater than that of product flavor (where we did not find a significant effect). Therefore, while flavour was more influential than receptacle colour as a whole, these two factors influenced disparate ratings in the present study. Intriguingly, there is a stronger association between carbonation and receptacle colour than between carbonation and product flavour, possibly because red colour and carbonation are both perceived to be higher in terms of arousal/excitement [29,47] whereas there are no clear associations between different flavours and carbonation. However, further research should be conducted in order to give a better view of the relative importance of those factors that affect perception and preference for consumer products such as food and beverages.

## 5. Conclusions

The present study found that for three commercially available, differently-flavoured carbonated beverages, the flavour as well as the receptacle colour generally affected consumer perceptions and preferences. The raspberry-flavoured drink was the most highly liked, and was rated as tasting the sweetest and least bitter, even though all three beverage flavours contained almost equal amounts of sugar (5.2 ± 0.1 g/100 mL, which is not likely to be perceptibly distinguishable [42]). In terms of receptacle colour, there was a significant influence on perceived carbonation, where beverages in red receptacles were rated as being more carbonated than beverages in black receptacles. Moreover, the influence of receptacle weight on perceived carbonation was moderated by the bitterness of the beverage. The results highlight the importance of understanding how food-intrinsic and extrinsic factors work together to form our overall perception and liking of food and beverages. This synergy is a key direction in perception science, and further research within this area is needed in order to approach more realistic consumption situations. Furthermore, with the increasing importance of designing healthier food and beverages, studies such as this one highlight the importance of crossovers between Research & Development on the one hand, and marketing/branding on the other hand, in order to increase the potential for success on the market.

## Figures and Tables

**Table 1:** Age and gender distribution of the participants in the three test groups.

	Group 1 (Grapefruit)	Group 2 (Raspberry)	Group 3 (Lemon)
*N*	134	132	135
Female	86	90	100
Age (Stdev)	33.0 (14.6)	32.8 (14.2)	30.6 (14.2)

**Table 2:** Nutritional contents of the three flavour variants of Tourtel^®^ Twist.

Flavour Variant	Grapefruit	Lemon	Raspberry
Energy (kcal/100 mL)	31	32	36
Carbohydrates (g/100 mL)	7.7	7.7	8.1
Sugars (g/100 mL)	5.1	5.1	5.3
Protein (g/100 mL)	0.2	0.4	0.2
Fat (g/100 mL)	0	0	0.1

**Table 3:** Pearson’s correlation coefficients between different product ratings.

	Liking	Sweetness	Sourness	Bitterness	Carbonation
**Liking**	1.0	0.39 **	−0.17 **	−0.29 **	−0.05 *
**Sweetness**	-	1.0	−0.18 **	−0.21 **	0.02
**Sourness**	-	-	1.0	0.39 **	0.12 **
**Bitterness**	-	-	-	1.0	0.15 **
**Carbonation**	-	-	-	-	1.0

* Indicates significance at the 0.05 level, and ** indicates significance at the 0.01 level.

**Table 4:** Effects of flavour, colour of receptacle, weight of receptacle, and their interactions for each rating category, in terms of the degrees of freedom, error degrees of freedom, *F* value, *p* value, and effect size (partial eta squared).

	Effect	*Df*	*Error df*	*F*	*p*	*η*_p_^2^
**Liking**	**Flavour**	**2**	**382**	**6.93**	0.001	**0.035**
	***Colour***	***1***	***382***	***3.21***	***0.07***	***0.008***
	Weight	1	382	1.59	0.21	0.004
	Colour × flavour	2	382	0.49	0.61	0.003
	Weight × flavour	2	382	0.27	0.77	0.001
	Colour × weight	1	382	0.18	0.68	<0.0005
	Colour × weight × flavour	2	382	0.60	0.55	0.003
**Sweetness**	**Flavour**	**2**	**382**	**10.24**	**<0.0005**	**0.051**
	Colour	1	382	2.36	0.13	0.006
	Weight	1	382	0.02	0.91	<0.0005
	Colour × flavour	2	382	0.93	0.40	0.005
	Weight × flavour	2	382	0.39	0.68	0.002
	Colour × weight	1	382	2.71	0.10	0.007
	Colour × weight × flavour	2	382	2.26	0.11	0.012
**Sourness**	Flavour	2	382	0.99	0.37	0.005
	Colour	1	382	0.004	0.95	<0.0005
	Weight	1	382	0.09	0.76	<0.0005
	Colour × flavour	2	382	0.25	0.78	0.001
	Weight × flavour	2	382	1.27	0.28	0.007
	Colour × weight	1	382	0.36	0.55	0.001
	Colour × weight × flavour	2	382	0.49	0.61	0.003
**Bitterness**	**Flavour**	**2**	**382**	**17.97**	**<0.0005**	**0.086**
	Colour	1	382	0.29	0.59	0.001
	Weight	1	382	0.49	0.49	0.001
	Colour × flavour	2	382	0.17	0.84	0.001
	Weight × flavour	2	382	0.97	0.38	0.005
	Colour × weight	1	382	0.42	0.52	0.001
	Colour × weight × flavour	2	382	0.93	0.40	0.005
**Carbonation**	Flavour	2	382	0.57	0.57	0.003
	**Colour**	**1**	**382**	**5.49**	**0.02**	**0.014**
	Weight	1	382	0.77	0.38	0.002
	Colour × flavour	2	382	1.12	0.33	0.006
	Weight × flavour	***2***	***382***	***2.76***	***0.06***	***0.014***
	Colour × weight	1	382	1.21	0.27	0.003
	Colour × weight × flavour	2	382	1.21	0.30	0.006

Significant effects (*p* < 0.05) are shown in bold. Trending effects (*p* < 0.10) have been italicised.
